# Hemolysis dictates monocyte differentiation via two distinct pathways in sickle cell disease vaso-occlusion

**DOI:** 10.1172/JCI172087

**Published:** 2023-09-15

**Authors:** Yunfeng Liu, Shan Su, Sarah Shayo, Weili Bao, Mouli Pal, Kai Dou, Patricia A. Shi, Banu Aygun, Sally Campbell-Lee, Cheryl A. Lobo, Avital Mendelson, Xiuli An, Deepa Manwani, Hui Zhong, Karina Yazdanbakhsh

**Affiliations:** 1 Laboratory of Complement Biology,; 2 Laboratory of Immune Regulation, and; 3 Clinical Research in Sickle Cell Disease, New York Blood Center, New York, New York, USA.; 4 Cohen Children’s Medical Center, New Hyde Park, Donald and Barbara Zucker School of Medicine at Hofstra/Northwell, Hempstead, New York, USA.; 5 Department of Pathology, University of Illinois at Chicago, Chicago, Illinois, USA.; 6 Laboratory of Blood-Borne Parasites,; 7 Laboratory of Stem Cell Biology and Engineering, and; 8 Laboratory of Membrane Biology, New York Blood Center, New York, New York, USA.; 9 Department of Pediatrics, Montefiore Medical Center, Albert Einstein College of Medicine, Children’s Hospital at Montefiore, New York, New York, USA.

**Keywords:** Hematology, Monocytes

## Abstract

Sickle cell disease (SCD) is a hereditary hemoglobinopathy characterized by painful vaso-occlusive crises (VOC) and chronic hemolysis. The mononuclear phagocyte system is pivotal to SCD pathophysiology, but the mechanisms governing monocyte/macrophage differentiation remain unknown. This study examined the influence of hemolysis on circulating monocyte trajectories in SCD. We discovered that hemolysis stimulated CSF-1 production, partly by endothelial cells via Nrf2, promoting classical monocyte (CMo) differentiation into blood patrolling monocytes (PMo) in SCD mice. However, hemolysis also upregulated CCL-2 through IFN-I, inducing CMo transmigration and differentiation into tissue monocyte–derived macrophages. Blocking CMo transmigration by anti–P selectin antibody in SCD mice increased circulating PMo, corroborating that CMo-to–tissue macrophage differentiation occurs at the expense of CMo-to–blood PMo differentiation. We observed a positive correlation between plasma CSF-1/CCL-2 ratios and blood PMo levels in patients with SCD, underscoring the clinical significance of these two opposing factors in monocyte differentiation. Combined treatment with CSF-1 and anti–P selectin antibody more effectively increased PMo numbers and reduced stasis compared with single-agent therapies in SCD mice. Altogether, these data indicate that monocyte fates are regulated by the balance between two heme pathways, Nrf2/CSF-1 and IFN-I/CCL-2, and suggest that the CSF-1/CCL-2 ratio may present a diagnostic and therapeutic target in SCD.

## Graphical abstract

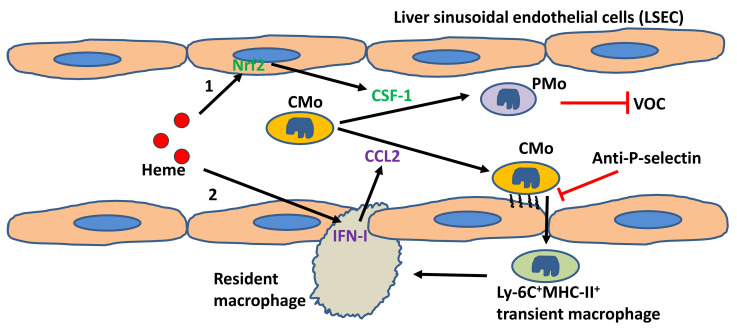

## Introduction

Sickle cell disease (SCD) is characterized by hemolytic anemia and painful vaso-occlusive crises (VOC), caused by increased adherence of sickle RBCs to the underlying activated vascular endothelium (1, 2) Accumulating evidence highlights the pivotal role of the mononuclear phagocyte system, encompassing blood monocytes, spleen red pulp macrophages, and liver Kupffer cells, in erythrophagocytosis of sickle RBCs and in the clearance of hemolytic byproducts in SCD (3–11). Our recent findings demonstrate that, a subset of blood monocytes, known as nonclassical monocytes or patrolling monocytes (PMo), are instrumental in mitigating VOCs in SCD by scavenging endothelial cell–attached (EC-attached) sickle RBCs and debris from hemolysis-damaged endothelium (12–14). In comparison with healthy donors (HD), patients with SCD exhibit reduced circulating PMo levels (12). During sickle crises, PMo frequency declines relative to steady state as a consequence of increased erythrophagocytosis of EC-bound sickle erythrocytes, overwhelming the compensatory PMo survival mechanisms and resulting in PMo death (13). However, the mechanisms underlying homeostatic regulation of circulating PMo numbers in SCD have not been clearly defined.

PMo primarily differentiate from classical monocytes (CMo) in the BM and circulation (15–17). Under normal conditions, CMo demonstrate a short circulatory life span, with the majority (90%–99%) transmigrating across the vascular endothelium into tissues, (18, 19) where they differentiate into tissue macrophages or dendritic cells (20–22). In contrast, a small proportion (1%–10%) of CMo follow an alternative differentiation pathway, becoming blood PMo, which, relative to CMo, have an extended life span and limited transmigration into tissues (18, 19). Nevertheless, the mechanisms regulating CMo differentiation into PMo versus migration and subsequent differentiation in tissue remain to be fully characterized.

In the context of SCD, despite a lower frequency of PMo, CMo numbers and levels of the CMo chemokine (chemokine[C-C motif] ligand 2 [CCL-2]), which promotes CMo migration to tissues, are increased (1, 3, 12, 22–24). Our recent study demonstrated an expansion of monocyte-derived macrophages in the liver and, to a lesser extent, in the spleen as a consequence of intravascular hemolysis (25). Based on these findings, we postulated and tested the hypothesis that hemolysis-induced CMo migration and differentiation into tissue macrophages in SCD occurs at the expense of blood CMo differentiation into PMo. A comprehensive understanding of SCD monocyte differentiation could provide valuable insights into the disease pathophysiology, including VOC, and inform the development of strategies to manipulate monocyte numbers to prevent disease progression in SCD.

## Results

### Hemolysis induces CSF-1 production in SCD.

**Figure 1:**
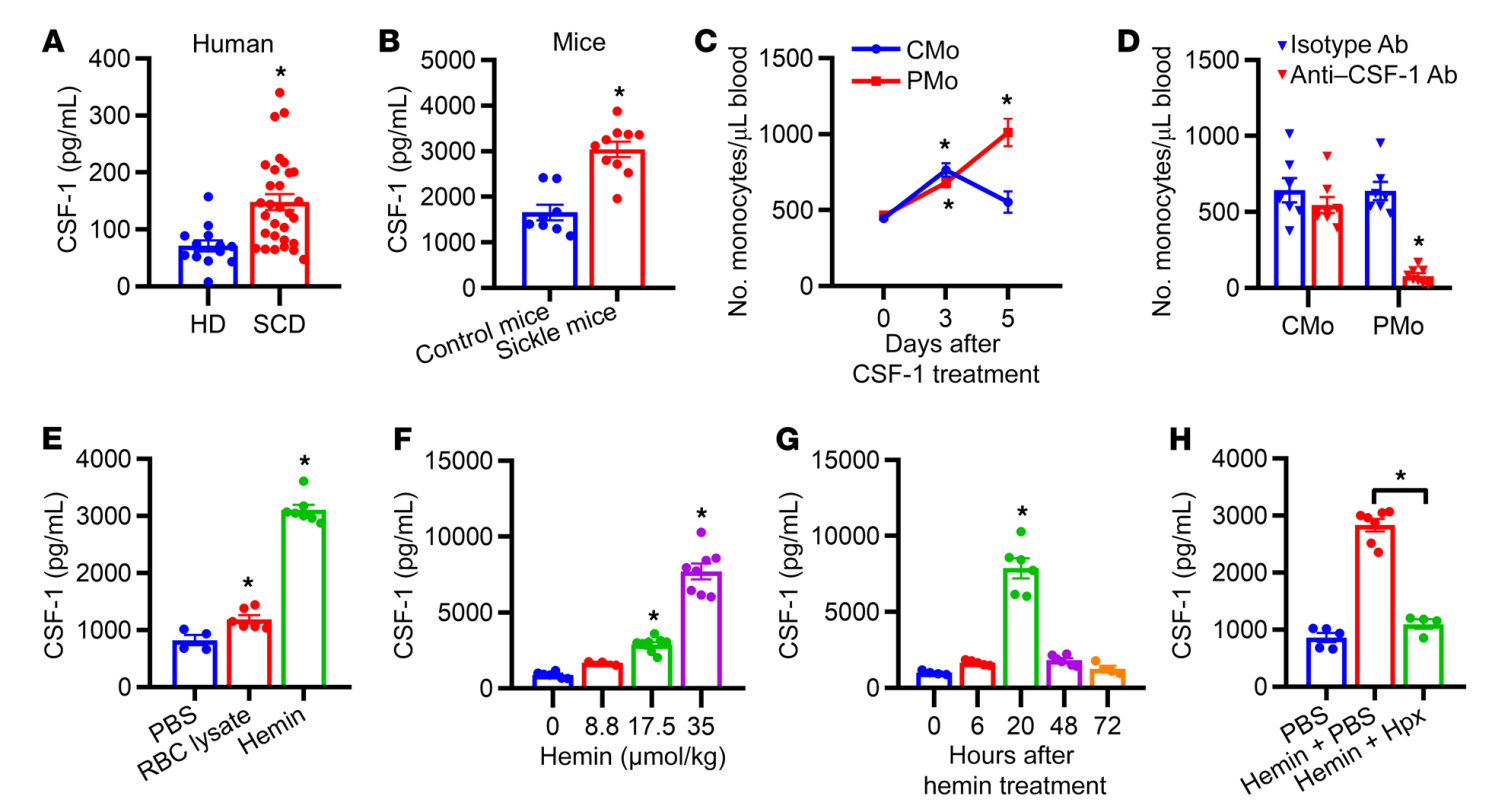
Assessment of plasma CSF-1 levels and monocyte numbers in SCD. (**A**) Plasma CSF-1 levels in HD (*n* = 13) and patients with SCD at steady state (*n* = 30). (**B**) Plasma CSF-1 levels in control and sickle mice (*n* = 8–10). (**C**) The absolute number of circulating Ly-6C^hi^ CMo and Ly-6C^lo/–^ PMo in sickle mice (*n* = 6–8) on day 3 and 5 after s.c. injection with CSF-1 (0.5 mg/kg body weight/d). (**D**) The absolute number of circulating Ly-6C^hi^ CMo and Ly-6C^lo/–^ PMo in sickle mice (*n* = 7–8) at 72 hours following i.p. injection with anti–CSF-1 blocking antibody (1 mg/kg body weight) or isotype antibody (1 mg/kg body weight). (**E**) Plasma CSF-1 levels in WT mice at 20 hours after i.v. injection of PBS as control (200 μL/20 g body weight), RBC lysate (17.5 μmol hemoglobin/kg body weight), or hemin (17.5 μmol/kg body weight) (*n* = 4–7). (**F**) Plasma CSF-1 levels in WT mice at 20 hours after i.v. injection with hemin at doses of 0, 8.8, 17.5, or 35 μmol/kg body weight (*n* = 3–9). (**G**) Plasma CSF-1 levels in WT mice at time points of 0, 6, 20, and 72 hours after i.v. injection with hemin (35 μmol/kg body weight) (*n* = 4–6). (**H**) Plasma CSF-1 levels in WT mice 20 hours after i.v. injection with PBS and hemin (17.5 μmol/kg body weight), hemin and hemopexin (17.5 μmol/kg body weight), or PBS alone (200 μL/20 g body weight) as control (*n* = 4–7). Each symbol represents data from an individual mouse. Data are shown as the mean ± SEM and were compared using a 2-tailed Student’s *t* test in **A**, **B**, and **D**; 2-way ANOVA with Bonferroni’s multiple comparisons in **C**; and 1-way ANOVA with Bonferroni’s multiple comparisons in **E**–**H**. *P < 0.05.

Monocytosis is a characteristic feature of SCD (3). Because colony-stimulating factor-1 (CSF-1) is a crucial growth factor for monocyte survival and differentiation, (26, 27), we measured and found elevated circulating CSF-1 in patients with SCD compared with that in HD (Figure 1A, 2-fold, *P* < 0.05, Supplemental Table 1; supplemental material available online with this article; https://doi.org/10.1172/JCI172087DS1) and in the Townes mouse model of SCD (sickle mice) relative to that in control mice (Figure 1B, 1.7-fold, *P* = 0.002). Injection of recombinant CSF-1 led to a 2-fold increase in the numbers of blood CMo on day 3 and PMo on day 5 in sickle mice (Figure 1C, *P* < 0.05, see gating strategies in Supplemental Figure 1A) and, as previously demonstrated in primates, including humans, a similar change in WT mice (Supplemental Figure 1B, *P* < 0.05) (28, 29). Blood neutrophil number, RBC number, and hemoglobin level in sickle mice did not show a change after CSF-1 treatment (Supplemental Figure 1, C–E). In contrast, treatment with a blocking anti–CSF-1 antibody in sickle mice resulted in a significant 8-fold reduction in circulating PMo numbers and a trend toward a decrease in circulating CMo (Figure 1D, compared with isotype antibody control), as reported in WT mice (30). Moreover, treatment with muramyl dipeptide (MDP), a known PMo inducer (31), led to increased plasma CSF-1 levels in WT mice, while pretreatment with anti–CSF-1 antibodies attenuated PMo expansion induced by MDP (Supplemental Figure 1, F and G, *P* < 0.05). To investigate whether hemolysis, a hallmark of SCD (32), can upregulate CSF-1 production, we induced acute hemolysis in WT mice using RBC breakdown products. Following injection of RBC lysate or hemin, plasma CSF-1 levels increased by 50% and 4-fold, respectively (Figure 1E, *P* < 0.05, compared with PBS control). We observed a dose-dependent increase in CSF-1 induction with escalating concentrations of hemin (Figure 1F, *P* < 0.05). Plasma CSF-1 was first detectable at 6 hours after hemin injection, peaked at 20 hours, and returned to baseline levels at 72 hours (Figure 1G, *P* < 0.05). Administration of hemin with heme scavenger hemopexin in WT mice completely abrogated the increase in plasma CSF-1 (Figure 1H, *P* < 0.05), substantiating the role of hemin in CSF-1 induction. Collectively, these findings suggest that intravascular hemolysis can trigger CSF-1 production and that CSF-1 is a key regulator of PMo numbers in SCD.

### Hemin-induced CSF-1 is produced partly by ECs through Nrf2 pathway.

**Figure 2:**
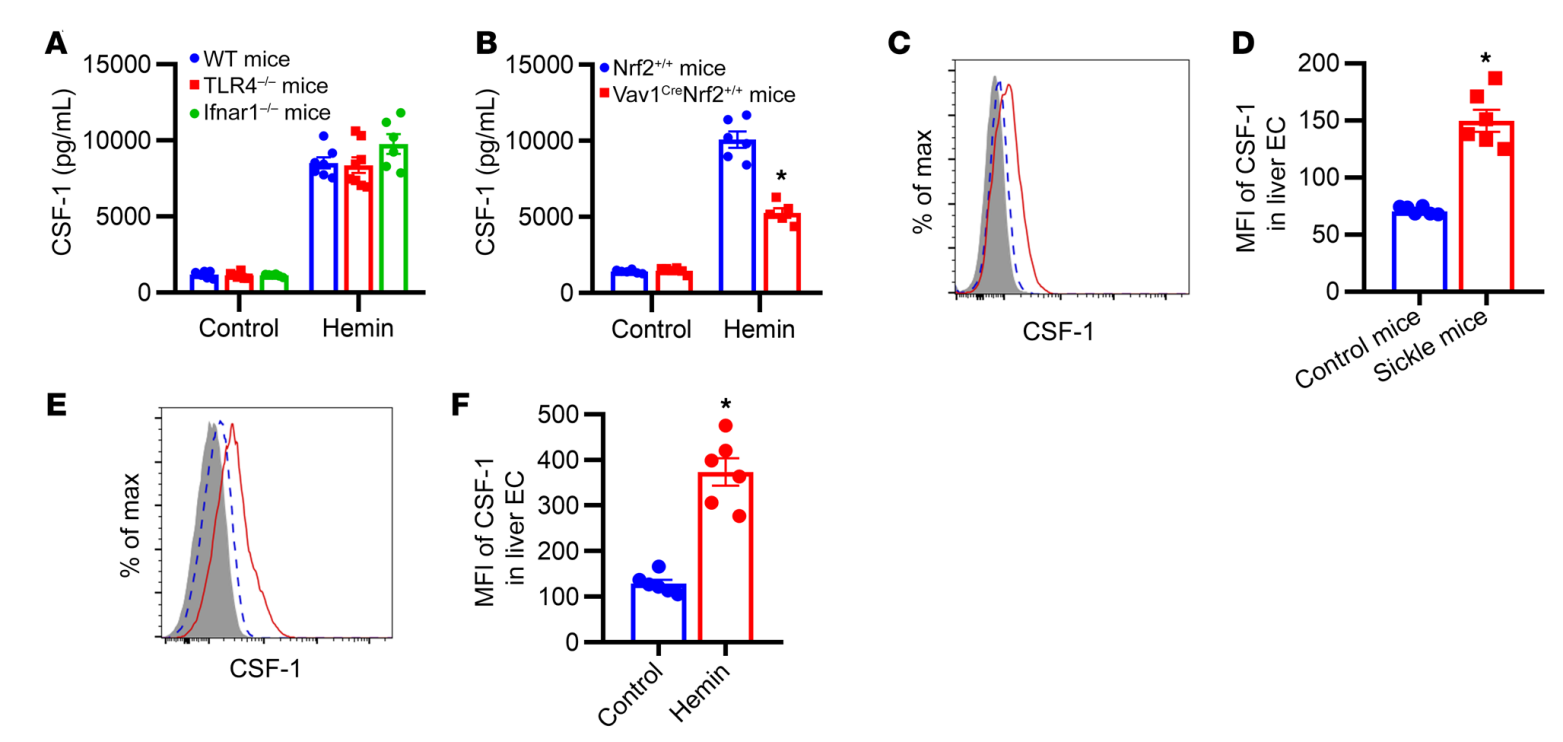
The mechanism of CSF-1 induction by hemolysis. (**A**) Plasma CSF-1 levels in WT mice, TLR4^–/–^ mice, and Ifnar1^–/–^ mice at 20 hours after i.v. injection with hemin (35 μmol/kg body weight) (*n* = 6–8). (**B**) Plasma CSF-1 levels in Vav1^cre^Nrf2^+/+^ mice and control Nrf2^+/+^ mice at 20 hours after i.v. injection with hemin (35 μmol/kg body weight) (*n* = 5–6). (**C**) Representative histogram and (**D**) bar graph showing CSF-1 expression in liver EC from control mice (blue dashed line, *n* = 6) and sickle mice (red solid line, *n* = 6). Isotype control is shown as the gray-filled histogram. (**E**) Representative histogram and (**F**) bar graph showing CSF-1 expression in liver EC from control WT mice (blue dashed line, *n* = 6) and hemin-treated WT mice (red solid line, *n* = 6). Gray-filled histogram represents the isotype control. Each symbol represents data from an individual mouse. Data are shown as the mean ± SEM and were compared using a 2-tailed Student’s *t* test. *P < 0.05.

We next investigated the role of key signaling pathways implicated in CSF-1 induction by hemolysis. Given that cell-free heme can activate TLR4 (33, 34) and type I IFN (IFN-I) pathways (25), we initially assessed the effect of hemin on the upregulation of plasma CSF-1 in TLR4^–/–^ and ifnar1^–/–^ mice. Interestingly, comparable CSF-1 levels were observed following hemin treatment in both gene knockout mouse strains and in WT mice (Figure 2A). Heme is also known to activate the nuclear factor erythroid 2–related factor 2 (Nrf2) transcription factor, which upregulates a broad array of antioxidant enzymes that protect against hemolysis (35). To evaluate the involvement of the heme/Nrf2 pathway in CSF-1 induction, we used Vav1-cre^+^Nrf2floxp^+/+^(Vav1^cre^Nrf2^+/+^) mice, which lack Nrf2 in the hematopoietic lineage and ECs (36). In these mice, we observed a 50% reduction in hemin-induced plasma CSF-1 levels compared with that in Nrf2^+/+^ control mice (Figure 2B, *P* < 0.05). To identify the cellular origin of CSF-1 in SCD, we analyzed CSF-1 protein levels using flow cytometry (37). Concentrating on the hepatic tissue, which serves as the primary heme detoxification organ (38), we observed elevated CSF-1 signals in liver ECs of sickle mice compared with control mice (Figure 2, C and D, 2-fold, *P* < 0.05; see gating strategies in Supplemental Figure 2A) and in hemin-treated WT mice relative to PBS-treated mice (Figure 2, E and F, 2.7-fold, *P* < 0.05). Although low levels of CSF-1 were detected in macrophages, no significant differences were found between sickle and control mice or between hemin-treated and PBS-treated WT mice (Supplemental Figure 2, B and C, see gating strategies in Supplemental Figure 2A). Furthermore, CSF-1 signals were essentially undetectable in all circulating monocyte subsets of sickle mice (data not shown), in line with the minimal CSF-1 transcripts (5 fragments per kilobase per million mapped fragments [FPKM], compared with HLA-DR transcripts of 1892 FPKM) observed in RNA-Seq analysis of human SCD peripheral blood CMo (Supplemental Figure 2D) (25). Collectively, these findings suggest that ECs, but not monocytes/macrophages, produce CSF-1 in SCD in response to hemolysis through the Nrf2 pathway and independent of TLR4 or IFNαR signaling.

### CMo-to-PMo differentiation in response to hemolysis correlates with circulating CSF-1/CCL-2 ratios.

**Figure 3:**
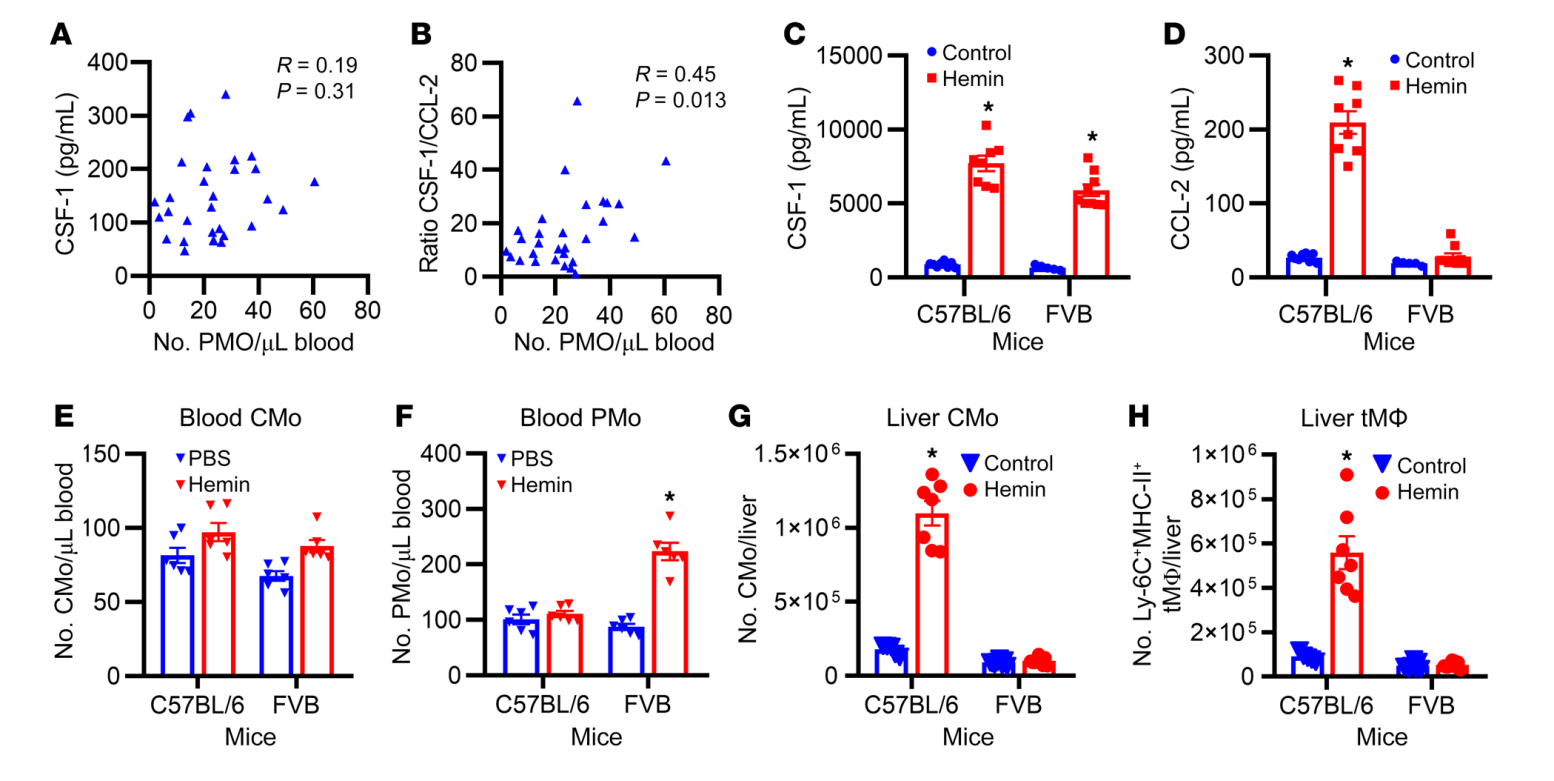
The relationship between PMo numbers and the ratio of CSF-1/CCL-2. (**A**) Scatter plot analysis showing correlation relationship between plasma CSF-1 levels and absolute numbers of circulating PMo in patients with SCD (*n* = 30, see Supplemental Figure 3A for human PMo gating strategy). (**B**) Scatter plot analysis showing correlation relationship between the ratio of plasma CSF-1 versus CCL-2 levels and absolute numbers of circulating PMo in patients with SCD (*n* = 30). (**C**) Plasma CSF-1 levels and (**D**) plasma CCL-2 levels in C57BL/6 mice and FVB mice 20 hours after i.v. injection with hemin (17.5 μmol/kg body weight) or PBS (200 μL/20 g body weight) as control (*n* = 5–9). (**E** and **F**) Absolute number of circulating Ly-6C^hi^ CMo (**E**) and Ly-6C^lo/–^ PMo (**F**) in mice at the time point of 3 days after injection, as shown in **C** (*n* = 6). (**G** and **H**) Absolute numbers of liver Ly-6C^hi^MHC-II^–^ CMo (**G**) and Ly-6C^+^MHC-II^+^ transient macrophages (tMΦ) (**H**) in mice injected as in **C** (*n* = 5–9). The correlation analysis in **A** and **B** was determined by Spearman’s Rho. Each symbol represents data from an individual mouse. Data are shown as the mean ± SEM and were compared using a 2-tailed Student’s *t* test. *P < 0.05.

To establish the relationship between CSF-1 and PMo numbers in patients with SCD, we conducted correlation analyses, but surprisingly, no association was found (Figure 3A, R = 0.19, *P* = 0.31; see gating strategies in Supplemental Figure 3A), suggesting a role for other factors that may affect CSF-1 control of PMo numbers in SCD. We have previously demonstrated that hemolysis results in upregulation of IFN-α–induced CCL-2 and subsequent CMo migration and differentiation into tissue monocyte–derived macrophages (25). Although PMo numbers did not directly correlate with CCL-2 levels (Supplemental Figure 3B), we found a significant positive correlation in patients with SCD between PMo numbers and the CSF-1/CCL-2 ratio (Figure 3B, R = 0.45, *P* = 0.013). These data suggest that the balance between CSF-1 and CCL-2 regulates blood PMo numbers in SCD. Interestingly, we observed a differential response to hemin in the induction of CSF-1 and CCL-2 in 2 mouse strains, WT C57BL/6 mice and FVB mice. Both mouse strains showed a 9-fold upregulation of circulating CSF-1 levels (Figure 3C, 1-day after hemin treatment; *P* < 0.05), but only C57BL/6 mice, but not FVB mice, showed increases in IFN-α (Supplemental Figure 3C) and CCL-2 (Figure 3D, approximately 8-fold, *P* < 0.05). Importantly, an increase in circulating PMo numbers was only detected in FVB mice (high CSF-1/CCL-2 ratio) but not in C57/BL/6 mice (Figure 3, E and F, day 3 after injection; *P* < 0.05). In contrast, elevated liver CMo and monocyte-derived macrophages (Ly-6C^+^MHC-II^+^ transient macrophage) were exclusively found in C57BL/6 mice (low CSF-1/CCl-2 ratio) (Figure 3, G and H, see gating strategies in Supplemental Figure 2A). Additionally, we found that MDP treatment resulted in a significant upregulation of CSF-1 but not CCL-2 in sickle mice, accompanied by an increase in circulating PMo numbers (13) but not liver CMo and Ly-6C^+^MHC-II^+^ transient macrophages (Supplemental Figure 3, D–F). Blood neutrophil number, RBC number, and hemoglobin level in sickle mice did not show a change after MDP treatment (Supplemental Figure 3, G–I). These findings suggest that, in response to hemolysis, a higher upregulation of CSF-1 along with low induction of CCL-2 promotes CMo-to-PMo differentiation in circulation, thus increasing blood PMo numbers, as observed in FVB mice. Conversely, if CCL-2 is also adequately induced, as observed in C57BL/6 mice or sickle mice, the outcome is tissue migration and differentiation of CMo into monocyte-derived macrophages instead of differentiation to PMo in blood.

### Blockade of CMo migration increases circulating PMo but reduces liver monocyte–derived macrophages.

**Figure 4:**
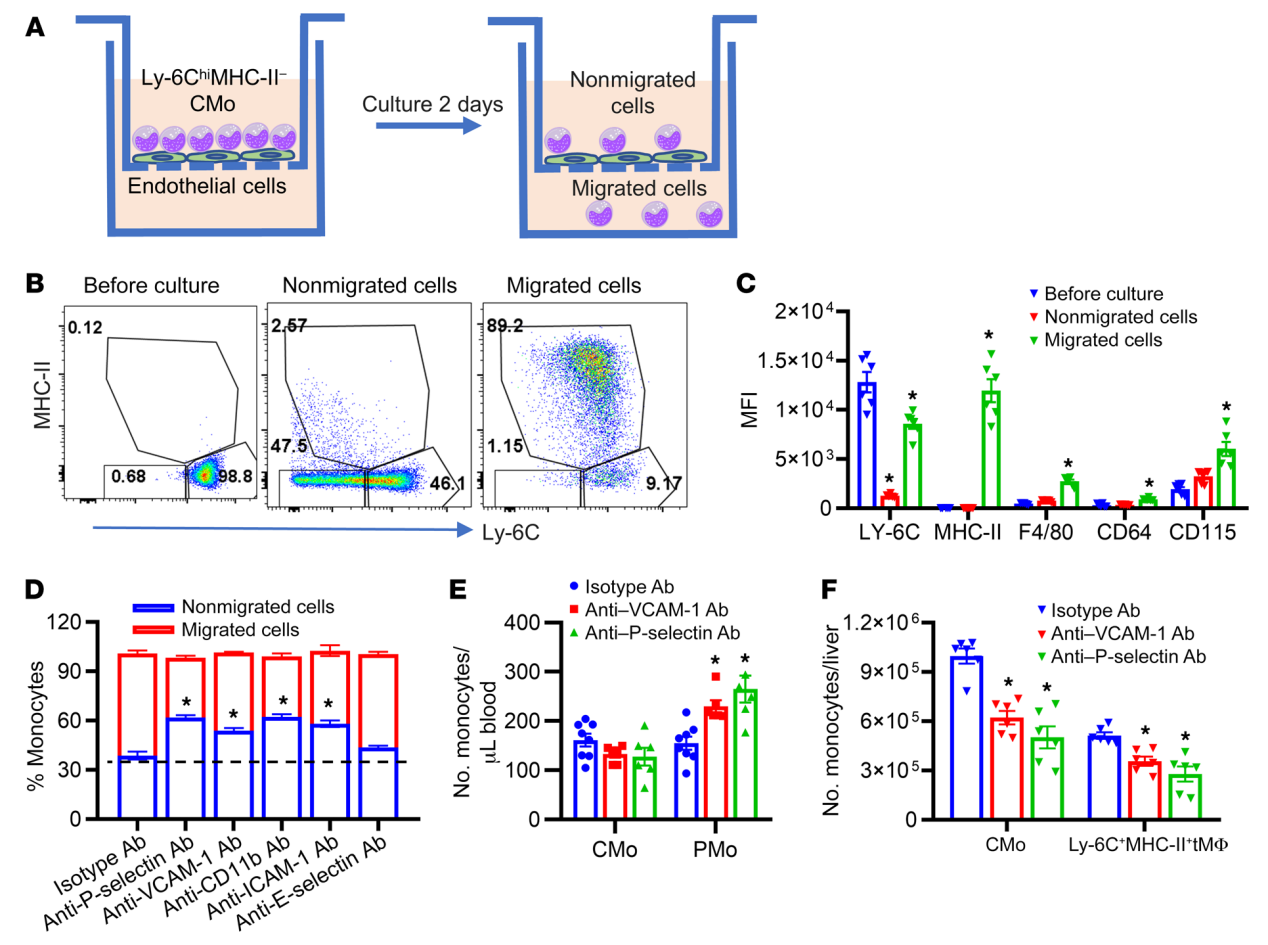
CMo migration blockade promotes differentiation into PMo in vitro and in vivo. (**A**) Schematic representation of experimental design. Transwell culture of purified BM Ly-6C^hi^MHC-II^–^ CMo placed above mouse endothelial cells (ECs) seeded in the upper compartment for 2 days. Monocytes in the bottom compartment are considered as having transmigrated through the ECs, while the ones remaining in the top well are considered the nonmigrated subpopulation. (**B**) Representative histograms showing the gating strategy for Ly-6C^hi^MHC-II^–^ CMo, Ly-6C^lo/–^MHC-II^–^ PMo, and Ly-6C^+^MHC-II^+^ transient macrophages in the Transwell culture, as shown in **A**. Numbers represent the frequency of Ly-6C^hi^MHC-II^–^ CMo (98.8, 46.1, and 9.17 before culture, nonmigrated cells, and migrated cells, respectively), Ly-6C^lo/–^MHC-II^–^ PMo (0.68, 47.5, and 1.15 before culture, nonmigrated cells, and migrated cells, respectively), and Ly-6C^+^MHC-II^+^ transient macrophages (0.12, 2.67, and 89.2 before culture, nonmigrated cells, and migrated cells, respectively). (**C**) Bar graph showing expression of surface markers on cultured monocytes, as shown in **A** (*n* = 6). (**D**) Bar graph showing the monocyte numbers in cocultures of purified BM Ly-6C^hi^MHC-II^–^ CMo layered above mouse ECs, which had been pretreated with blocking antibody against P selectin, VCAM-1, ICAM-1, E selectin, CD11b, or isotype control (10 ng/mL, *n* = 6). (**E**) Bar graph showing absolute number of circulating Ly-6C^hi^ CMo and Ly-6C^lo/–^ PMo at 20 hours in hemin-injected (35 μmol/kg body weight) WT mice pretreated for 30 minutes with anti–P selectin blocking antibody (5 mg/kg body weight, i.v.), anti–VCAM-1 blocking antibody (5 mg/kg body weight, i.v), or isotype control antibody (5 mg/kg body weight, i.v) (*n* = 6). (**F**) Bar graph showing absolute numbers of liver Ly-6C^hi^MHC-II^–^ CMo and Ly-6C^+^MHC-II^+^ transient macrophages (tMΦ) in mice, injected as in **D** (*n* = 6). Each symbol represents data from an individual mouse. Data are shown as the mean ± SEM and were compared using 2-way ANOVA with Bonferroni’s multiple comparisons. *P < 0.05.

To establish that transmigration and differentiation of CMo into macrophages occurs at the expense of CMo-to–blood PMo differentiation, we employed an in vitro Transwell culture model wherein purified mouse CMo, identified as Ly-6C^+^MHC-II^–^ cells, were placed in the upper chamber of the Transwell, which was preseeded with ECs to simulate CMo transendothelial migration (39). Monocytes that migrated across ECs into the bottom well exhibited MHC-II expression (Figure 4, A–C), implying their predisposition toward Ly-6C^+^MHC-II^+^ transient macrophage differentiation as previously reported (39). Interestingly, monocytes that did not migrate but remained in the top well did not display elevated MHC-II expression (Figure 4, A–C). Instead, they exhibited a characteristic PMo-like phenotype, marked by decreased Ly-6C levels and lower expression of macrophage markers, such as F4/80, CD64, and CD115, compared with migrated cells (Figure 4C, *P* < 0.05). These data suggest that nonmigrated CMo on ECs can differentiate into PMo. Adhesion molecules play a critical role in CMo transendothelial migration. To examine whether inhibiting CMo transmigration across ECs through adhesion molecule blockade mediated increased PMo differentiation, we pretreated ECs in the Transwell culture system with blocking antibodies targeting key adhesion molecules, including P selectin, VCAM-1, CD11b, ICAM-1, E selectin, or isotype control. With every antibody except anti-E selectin antibody, we observed an increased frequency of nonmigrated cells (mostly PMo-like monocytes) and a concomitant decrease in migrated cells (mostly Ly-6C^+^MHC-II^+^ transient macrophages) (Figure 4D, *P* < 0.05), suggesting that impeding CMo transendothelial migration may promote PMo differentiation. We next tested whether these blocking antibodies had an effect on monocyte numbers in an in vivo hemolysis model. In hemin-injected WT mice pretreated with blocking antibodies against VCAM-1 or P selectin, compared with isotype control antibody, we found a 2-fold increase in blood PMo numbers along with a 50% decrease in liver CMo numbers and 20% reduction in Ly-6C^+^MHC-II^+^ transient macrophages (Figure 4, E and F, *P* < 0.05). Blocking antibodies against E selectin demonstrated no substantial efficacy in modulating monocyte migration and differentiation processes in this in vivo model (Supplemental Figure 4, A and B). Because we used blocking antibodies against CD11b and ICAM-1 caused depletion of monocyte/macrophages in this model (data not shown), we were unable to assess their role in CMo tissue migration. In nonhemolytic conditions, CMo can differentiate into PMo first and then differentiate into macrophages (40). To determine whether this differentiation pathway is also relevant within a hemolysis context, we administered hemin to both WT and Nr4a1-knockout mice (characterized by a lack of PMo) (41). However, the induction of liver CMo and Ly-6C^+^MHC-II^+^ transient macrophages following hemolysis was comparable in both WT and Nr4a1-knockout cohorts (Supplemental Figure 4, C and D), suggesting that the pathway for hemolysis-induced liver monocyte–derived macrophage differentiation is independent of PMo. Collectively, our findings demonstrate that obstructing CMo transendothelial hepatic migration, which is induced by hemolysis, can increase circulating PMo numbers, while reducing tissue monocyte–derived macrophages.

### CSF-1 plus anti–P selectin antibodies induce circulating PMo and reduce VOC in sickle mice.

**Figure 5:**
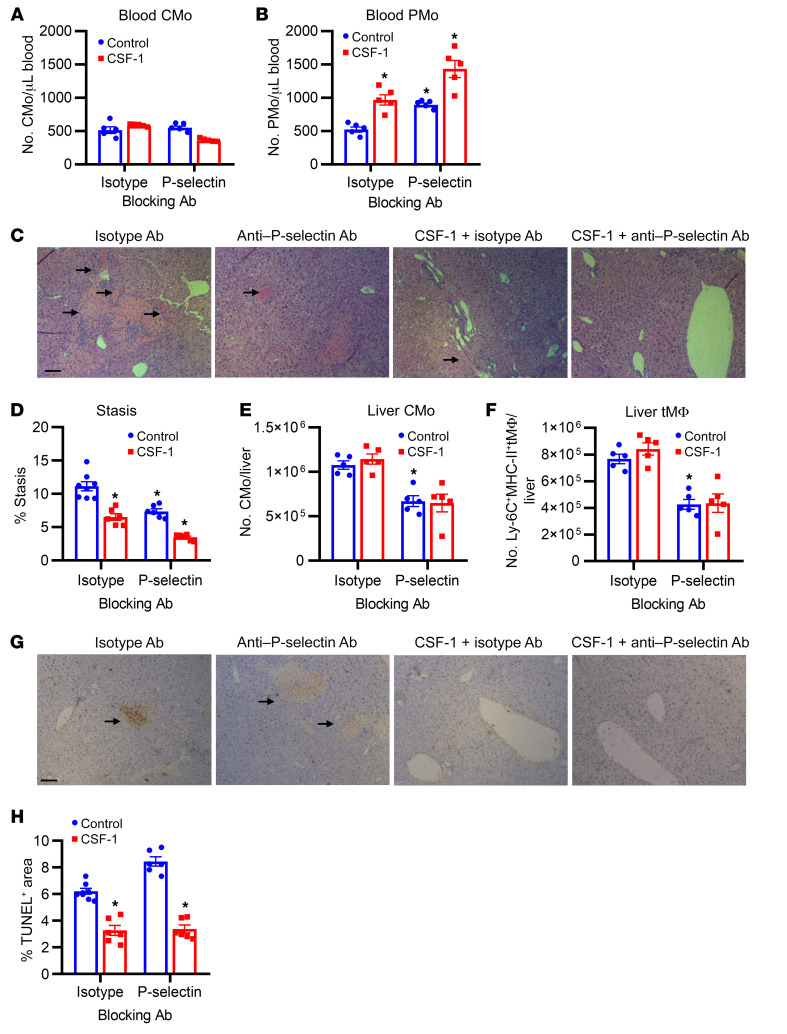
Effects of anti–P selectin blockade and CSF-1 treatment on monocyte subpopulations and liver pathology in sickle mice. (**A** and **B**) Bar graphs showing the absolute numbers of circulating Ly-6C^hi^ CMo (**A**) and Ly-6C^lo/–^ PMo (**B**) in sickle mice at day 5 after administration with anti–P selectin blocking antibody or isotype control antibody alone (5 mg/kg body weight, i.p. every other day) or with s.c. injection with CSF-1 (0.5 mg/kg body weight/d) (*n* = 5). (**C**) Representative H&E-stained liver sections from mice, injected as in **A** (scale bar: 100 μm). Black arrows indicate RBC stasis within blood vessels. (**D**) The frequency of occluded blood vessels (% stasis) in liver sections from mice injected as in **A** (*n* = 6–8). (**E** and **F**) Bar graphs showing the absolute numbers of liver Ly-6C^hi^MHC-II^–^ CMo (**E**) and Ly-6C^+^MHC-II^+^ transient macrophages (tMΦ) (**F**) in mice injected as in **A** (*n* = 5). (**G**) Representative TUNEL-stained liver sections from mice injected as in **A** (scale bar: 100 μm). Black arrows indicate TUNEL-positive areas. (**H**) The frequencies of necrotic areas (TUNEL positive) in liver sections from mice injected as in **A** (*n* = 6–8). Each symbol represents data from an individual mouse. Data are shown as the mean ± SEM and were compared using a 2-tailed Student’s *t* test. *P < 0.05.

We previously established that modulating PMo numbers in sickle mice can alter red cell stasis (13). We reasoned that CSF-1–induced PMo might prevent VOC and that a combination of CSF-1 and anti–P selectin antibody might even further enhance PMo numbers to thus improve VOC. To test this hypothesis, sickle mice were administered anti–P selectin or isotype control antibodies or a combination of CSF-1 and anti–P selectin or isotype control antibodies. Consistent with the data in Figure 1, CSF-1 combined with the isotype antibody led to a rise in circulating PMo numbers compared with the isotype antibody alone, whereas CSF-1 paired with anti–P selectin antibody resulted in even higher circulating PMo levels compared with each alone (Figure 5, A and B, *P* < 0.05). Histological (H&E) analysis of liver vasculature revealed diminished vascular stasis (by half) in sickle mice treated with CSF-1 and isotype antibody or anti–P selectin antibody alone compared with mice treated with isotype antibody, a finding that was further enhanced in mice treated with CSF-1 and anti–P selectin antibody (Figure 5, C and D, *P* < 0.05). Treatment with CSF-1 combined with the isotype antibody did not alter liver CMo and Ly-6C^+^MHC-II^+^ transient macrophage numbers in sickle mice; however, treatment with anti–P selectin antibodies (which block CMo migration) with or without CSF-1 resulted in decreases in these numbers (Figure 5, E and F, *P* < 0.05). Macrophages play a crucial role in protecting against liver damage in SCD (25, 42). Unlike Ly-6C^+^MHC-II^+^ transient macrophage numbers, we observed an increase in liver-resident macrophage (F4/80^hi^Tim-4^+^, see gating strategies in Supplemental Figure 2A) numbers following CSF-1 administration (Supplemental Figure 4E, *P* < 0.05), as in WT mice reported previously (43), but not in spleen macrophage numbers (Supplemental Figure 4F). However, anti–P selectin antibody had no effect on resident macrophage numbers (Supplemental Figure 4E). TUNEL staining of liver tissue revealed diminished liver injury in sickle mice treated with CSF-1 and isotype or anti–P selectin antibody compared with either antibody without CSF-1 (Figure 5, G and H). In mice treated with anti–P selectin antibody alone, liver injury was slightly increased (Figure 5, G and H), which is in line with recent data in P selectin–deficient mice (44). Altogether, these data suggest that, compared with either treatment alone, combination therapy with CSF-1 and anti–P selectin further increases PMo numbers and is more efficacious in prevention of VOC. Furthermore, CSF-1 drives expansion of macrophages that prevent liver damage.

## Discussion

In this study, we demonstrate that hemolysis regulates monocyte fate through two distinct pathways in SCD. Using the Townes SCD mouse model, we found that heme-induced production of CSF-1, primarily by tissue ECs via Nrf2, promotes the differentiation of CMo into PMo. On the other hand, heme-induced CCL-2 drives mouse blood CMo transmigration into tissues and their differentiation into monocyte-derived macrophages through IFN-I. We also found that the relative ratio of plasma CSF-1 and CCL-2 levels, both elevated in SCD, directly correlates with blood PMo numbers in patients with SCD, suggesting that the balance between CSF-1 and CCL-2 pathways dictates circulating monocyte fates under hemolytic conditions. Based on our mouse data that CMo-to-monocyte–derived macrophage differentiation occurs at the expense of blood PMo differentiation, we established an in vitro mouse EC culture system for expansion of PMo from CMo by inhibiting transmigration. Importantly, combination therapy with CSF-1 and anti–P selectin blocks monocyte transmigration into tissues, further bolstering PMo numbers and conferring better protection against stasis in SCD mice than either treatment alone.

A key finding of our study is that PMo numbers in SCD are determined by the CSF-1/CCL-2 ratio. It is well-established that CMo can be expanded by CSF-1 (45), migrate into tissue after induction by CCL-2 (22), and subsequently differentiate into macrophages or dendritic cells under both steady-state (46) and proinflammatory conditions (22). Nevertheless, the precise regulatory mechanism governing the differentiation of blood CMo into PMo under these circumstances remains unclear. MDP, a peptidoglycan motif common to all bacteria, injected i.v. has been shown to induce PMo production in various mouse models including SCD (13, 31, 47). Our data reveal that PMo expansion by i.v. MDP drives higher upregulation of blood CSF-1 compared with CCL-2. This is in contrast to a previous report showing that, in a bacterially induced Crohn’s disease model, MDP, which was produced in tissues, led to the upregulation of CCL-2 (48). We speculate that the response to i.v. exposure to MDP may be different from that to locally produced MDP, although in that report, neither CSF-1 levels nor the effect on circulating PMo numbers were measured (48). Our analysis centered on the roles of CSF-1 and CCL-2 on monocyte differentiation, because these two factors are upregulated in hemolytic conditions (hemin treatment) and SCD. Our observations particularly highlighted an increase in plasma CSF-1 levels that was mainly attributed to ECs, although a role for mesenchymal stromal cells/fibroblasts, which are also a major source of CSF-1, cannot be excluded (26, 49). Our findings have focused on the role of cell-free heme, but we do not necessarily preclude the potential involvement of other damage-associated molecular patterns released during intravascular hemolysis in the regulation of CSF-1 production in SCD (50). Other growth factors and chemokines may also contribute, such as CSF-2, which plays a crucial role in at least mouse monocyte development (51, 52). Interestingly, circulating levels of CSF-2 are reported to be elevated in patients with SCD (53, 54). However, administration of anti–CSF-2 blocking antibodies did not affect the numbers of blood PMo in sickle mice (446 ± 39/μL blood vs. 432 ± 48/μL blood with isotype antibody treatment), suggesting a CSF-2–independent regulation of blood PMo number in our mouse model. Additionally, CX3CL1 and its receptor CX3CR1 are crucial for PMo migration, although it remains controversial whether blood PMo numbers are reduced in Cx3CR1-knockout mice (55–57). Other chemokines, such as CCL7, CCL8, and CCL12, influence monocyte migration but are not considered as critical as CCL-2 and exhibit less monocyte specificity, as they also attract various other white blood cells (22). CCL2 plasma levels and PMo numbers/frequency are also altered in endotoxemia (58), myocardial infarction (59), and malaria (60). However, the levels of circulating CSF-1 in these conditions were not determined at the same time as CCL2 and/or PMo analysis. Thus, the relevance of the CSF-1/CCL2 ratio to blood PMo numbers in these or other diseases remains to be examined to ascertain whether the balance between CSF-1 and CCL-2 in regulating blood PMo numbers is unique to SCD or if this can be extended to other disease states. We observed a modest reduction in total monocyte numbers in patients on hydroxyurea compared with those not receiving the treatment (data not shown), which is consistent with previous reports (61, 62). However, we did not identify a statistically significant difference in the PMo numbers between the two groups (12). It has been shown that patients with SCD on hydroxyurea had higher frequency of PMo when compared with those receiving transfusions (63). We posit that hydroxyurea treatment leads to a reduction in circulating CMo numbers but, due to a compensatory mechanism to maintain total circulating monocyte numbers, PMo life span is extended (17) and, therefore, PMo frequency is increased in treated patients, although PMo numbers are not (12).

It is generally accepted that CMo do not differentiate into PMo when cultured in vitro (64–66). Numerous studies, including our own here, have shown that in the presence of ECs, CMo transmigrate and differentiate into macrophage-like cells (20, 39). We show that, under such culture conditions, nonmigrated cells adopt PMo-like phenotypes. Although the functional properties of these PMo-like cells remain to be further examined, our discoveries pave the way for devising novel protocols that utilize ECs as feeder cells for ex vivo PMo expansion, as a first step toward developing a cell therapy product. EC-mediated regulation of CMo-to-PMo differentiation could occur through direct EC-CMo interactions. For example, it has been shown that the interaction of EC-expressed Delta-like 1 (DLL1) with monocyte Notch2 receptor promotes CMo-to-PMo differentiation in vitro (67). However, the same group has also shown that DLL1 can induce CMo-to-macrophage differentiation in vitro (68), raising the possibility that DLL1/Notch2 signaling might not be specific for PMo differentiation. Alternatively, ECs may act as a barrier that prevents exposure to the unique extravascular tissue environment that otherwise supports CMo differentiation into monocyte-derived macrophages or even dendritic cells. Thus, CMo would differentiate into PMo in the bloodstream by a default pathway if they do not cross the ECs into tissues (69). However, it should be noted that hematopoietic organs such as BM and spleen are an exception, because they harbor specialized niche microenvironments that support CMo-to-PMo differentiation.

An exciting finding of our study is that coadministration of CSF-1 in conjunction with P selectin blockade augmented PMo numbers and diminished stasis in sickle mice more effectively than utilizing either anti–P selectin antibody or CSF-1 alone. To a lesser extent, P selectin blockade alone also increased blood PMo numbers and reduced stasis in sickle mice consistent with P selectin–knockout sickle mice exhibiting increased circulating monocytes and decreased liver myeloid cells, (44, 70), but the monocyte/macrophage populations in the latter mouse model have yet to be characterized (44). In our study, we found that CSF-1 plus anti–P selectin antibody–treated mice displayed reduced liver injury, possibly due to increased resident tissue macrophages in response to CSF-1 (Supplemental Figure 3G), which is known to promote tissue macrophage proliferation (71). This is in contrast to heightened liver tissue injury observed with anti–P selectin antibody treatment alone (Figure 5) or P selectin–knockout sickle mice (44). To date, no hepatic abnormalities have been reported in patients with SCD on crizanlizumab, although data on long-term use of crizanlizumab are as yet not available. These data raise a compelling rationale for concomitant use of CSF-1 and anti–P selectin antibody in SCD, because it could serve dual functions: mitigating vaso-occlusion by elevating circulating PMo numbers while simultaneously bolstering tissue-resident macrophage populations to protect against organ damage. Thus, we propose that the combined administration of crizanlizumab, a humanized anti–P selectin antibody licensed as a treatment option for VOC, and CSF-1 may yield even greater efficacy in SCD management. Mechanistically, we also propose that the ability of crizanlizumab to increase PMo numbers may serve as an additional mechanism of action for safeguarding against VOC.

In conclusion, our study utilizing a mouse model of SCD has pinpointed hemolysis as a key factor in SCD promoting CSF-1 production, leading to PMo expansion if CCL2, which promotes the monocyte-derived macrophage pathway, is relatively suppressed. We postulate that novel or existing therapies that increase the CSF-1/CCL2 ratio will be more efficacious in reducing pain. We thus propose that a heightened PMo number achieved through the manipulation of the CSF-1/CCL2 ratio may contribute to a reduced risk of VOC in SCD.

## Methods

### Human samples.

A cohort of patients were on chronic transfusion therapy (every 3–4 weeks for minimum of 2 years using leukodepleted units, phenotype matched for the C, E, and K red cell antigens), immediately pretransfusion. Race-matched control samples were obtained from deidentified volunteer HD at the New York Blood Center. All blood specimens underwent processing within 18 hours of collection. Patient clinical characteristics are detailed in Supplemental Table 1.

### Mice.

HbSS-Townes sickle mice (homozygous for β^S^) and HbAA-Townes control mice (homozygous for β^A^) were obtained by breeding HbAS-Townes mice (013071, The Jackson Laboratory). C57BL/6J WT mice (000664), Tlr4^–/–^ mice (029015), Ifnar1^–/–^ mice (028288), Nr4a1^–/–^ mice (006187), and FVB mice (001800) were acquired from The Jackson Laboratory. Vav1-cre^+^Nrf2floxp^+/+^(Vav1^cre^Nrf2^+/+^) mice, deficient in Nrf2 in the hematopoietic lineage and ECs, were obtained by crossing Vav1-cre mice (008610) with Nrf2floxp mice (025433), which were obtained from Larry Luchsinger (New York Blood Center) (72). All mice were bred in house, fed a standard rodent chow diet, and housed in microisolator cages in a special pathogen–free facility.

### Plasma preparation and cell isolation.

Whole blood samples were analyzed for complete blood counts and leukocyte differentials utilizing the Advia 120 Hematology Analyzer (Siemens Healthcare Diagnostics). For the isolation of PBMCs, human blood samples underwent centrifugation at 282*g* for 10 minutes. Subsequently, supernatants were removed for plasma preparation as previously described (73), and white cell pellets were subjected to density gradient centrifugation using Ficoll (GE Healthcare) to isolate PBMCs.

Mouse white blood cells were prepared from whole blood by lysing RBCs with RBC Lysis Buffer (Thermo Fisher Scientific). Plasma was obtained from the whole blood supernatant through centrifugation at 500*g* for 10 minutes. Mouse liver single-cell suspensions were generated using a liver dissociation kit (Miltenyi Biotec) in combination with the gentleMACS Octo Dissociator with Heaters (Miltenyi Biotec) according to the manufacturer’s guidelines. RBCs within the organ single-cell suspensions were lysed using RBC Lysis Buffer (Thermo Fisher Scientific).

### Antibodies, flow cytometry, and ELISA.

Fluorescently labeled anti-human antibodies against CD14 (M5E2), CD16 (3G8), HLA-DR (G46-6), and CD45 (HI30) were purchased from BD Biosciences. Human Fc blocker (human IgG) was purchased from Miltenyi Biotec. LIVE/DEAD Fixable Violet Dead Cell Stain (ViViD) and DAPI were obtained from Thermo Fisher Scientific. Fluorescently labeled mAbs anti-mouse CD45 (30-F11), CD11b (M1/70), CD64 (X54-5/7.1), F4/80 (BM8), Ly-6C (HK1.4), Ly-6G (1A8), CD11c (N418), MHC-II (M5/114.15.2), CD24 (M1/69), Tim-4 (RMT4-54), and anti-mouse CD16/32 (93, as Fc blocker) were purchased from Biolegend. Unconjugated anti-mouse CSF-1 polyclonal antibody (goat) was purchased from R&D Systems and conjugated using the Zenon labeling Kit (Thermo Fisher Scientific) before use.

For the assessment of marker expression in human and mouse specimens via flow cytometry, single cells were prepared and first stained with the dead cell marker ViViD, and after 2 washes, they were fixed/permeabilized with the intracellular fixation & permeabilization buffer set (Thermo Fisher Scientific) following the manufacturer’s instructions. Cells were incubated with Fc blocker and then stained with various fluorescent antibodies in different staining configurations. Data acquisition was performed on an LSR Fortessa flow cytometer (BD Biosciences), and the resulting data were analyzed utilizing the FlowJo software (Tree Star).

To quantify the plasma concentrations of CSF-1 in human and mouse samples, ELISA kits from R&D Systems and Biolegend, respectively, were used. For the evaluation of CCL2 plasma levels in both human and mouse specimens, the Cytometric Bead Array kit (BD Biosciences) was used, according to the provided manufacturer’s protocol.

### Hemin and RBC lysate preparation.

Hemin was purchased from Frontier Scientific and was dissolved in 0.2 M sodium hydroxide (NaOH), followed by neutralization to a pH of 7.2 using hydrochloric acid (HCl). For the preparation of RBC lysates, purified RBC pellets from WT mice were lysed in water (10-fold volume) at room temperature for 30 minutes. Cellular debris was then pelleted by centrifugation at 13,000*g* for 15 minutes. Supernatants were retained and supplemented with 10× PBS (1:10 volume). Total heme levels in the supernatants were tested using a QuantiChrom heme assay kit (BioAssay Systems).

### In vivo treatment.

To investigate the induction of CSF-1 in vivo, mice were injected i.v. with freshly prepared hemin (8.8–35 μmol/kg body weight), RBC lysate (17.5 μmol heme/kg body weight), or MDP (InvivoGen, 1 mg/kg body weight/d), while PBS (200 μL/20 g body weight) served as a control. In some experiments, hemin was combined with hemopexin at a 1:1 ratio. For the administration of exogenous CSF-1, mice were injected s.c. in the loose skin over the neck with recombinant human CSF-1 (0.5 mg/kg body weight/d, PeproTech) or PBS as a control for 4 consecutive days. To block endogenous CSF activity, mice were injected i.p. with a blocking antibody against CSF-1 (5A1), CSF-2 (MP1-22E9), or isotype control (1 mg/kg body weight, Bio X Cell). For blockade of CMo migration in vivo, mice were treated with Ultra-LEAF (Low Endotoxin, Azide-Free) blocking antibodies against CD62p (RB40.34, BD), CD106 (M/K-2.7, Bio X Cell), E selectin (9A9, Bio X Cell), CD11b (M1/70, Biolegend), ICAM-1 (YN1/1.7.4, Biolegend), or isotype control (Bio X Cell) (5 mg/kg body weight, i.v.). Mice were sacrificed at various time points after treatment, and blood samples and liver tissues were collected for subsequent analysis.

### In vitro coculture of CMo and EC within Transwell system.

CMo were purified from WT mice BM using negative selection with a cocktail of biotin-labeled antibodies against CD19 (6D5), CD3 (145-2C11), TER-119 (TER-119), Ly-6G (1A8), NK1.1 (PK136), CD170 (M1305A02), CD11c (N418), and MHC-II (M5/114.15.2) (all from Biolegend), followed by anti-biotin microbeads (Miltenyi) according to the manufacturer’s instructions. The in vitro coculture experiments were conducted using 6.5 mm Transwell inserts with 3.0 μm pore polycarbonate membrane and CellAdhere Collagen I–Coated 24-Well Flat-Bottom Plates (Stem Cell). Purified CMo were added onto the insert precultured with confluent mouse EC line bEnd.3 [BEND3] (ATCC). The coculture was evaluated after a 48-hour incubation period. To block CMo migration, Ultra-LEAF (Low Endotoxin, Azide-Free) blocking antibodies against P selectin (RB40.34, BD), VCAM-1 (M/K-2.7, Bio X Cell), E selectin (9A9, Bio X Cell), CD11b (M1/70, Biolegend), ICAM-1 (YN1/1.7.4, Biolegend), or isotype control (Bio X Cell) were added 30 minutes before initiating the coculture at a concentration of 10 ng/mL.

### Histological analysis.

Histological and immunohistochemical assessments were conducted by the Laboratory of Comparative Pathology at Weill Cornell Medicine/Memorial Sloan Kettering Cancer Center. Tissue samples were fixed in formalin, embedded in paraffin, and sectioned at a thickness of 5 μm. The sections were stained with H&E (Sigma-Aldrich) or TUNEL according to standard procedures and examined using a Leica DM 2000 microscope.

### Statistics.

The data in each experiment were analyzed separately and are displayed as individual data points in figures. Data are shown as the mean ± SEM. Statistical analyses were performed using GraphPad Prism. To determine the statistical significance of the differences between experimental groups, a 2-tailed Student’s *t* test was used. A *P* value of less than 0.05 was considered statistically significant.

### Study approval.

All mouse experiments were approved by the New York Blood Center’s Animal Care and Use Committee. All human studies were approved by the Institutional Review Boards of the New York Blood Center, Montefiore Medical Center, Feinstein Institutes for Medical Research/Northwell Health (Manhasset, New York, USA), and University of Illinois at Chicago. Blood samples were obtained after informed consent was received from patients with SCD, all homozygous for hemoglobin S.

### Data availability.

All data supporting the findings of this study are available within the article and supplemental materials. Values for all data points in graphs are reported in the Supporting Data Values file. The RNA-Seq data have been published (25) and are accessible in the in Gene Expression Omnibus (GEO GSE168532).

## Authorship contributions

YL conceived the idea, performed experiments, and analyzed the data. S Su, S Shayo, WB, MP, and KD performed experiments, and analyzed the data. XA, CAL, AM, and PAS assisted with project concept and design. PAS, BA, SCL, and DM were involved with all aspects of selection, recruitment, and provision of blood samples from patients and controls. HZ analyzed and interpreted data. KY directed the overall research design, and project supervision. KY and YL wrote the manuscript with consultation and contribution from all coauthors.

## Supplementary Material

Supplemental data

Supporting data values

## Version 1 07/25/2023

In-Press Preview

## Version 2 09/15/2023

Electronic publication
