# Supplementary material for: Hemolysis dictates monocyte differentiation via two distinct pathways in sickle cell disease vaso-occlusion
Source: J Clin Invest. 2023 Sep 15;133(18):e172087. doi: 10.1172/JCI172087 (PMC10503794; doi:10.1172/JCI172087)
Supplement: Supplemental data [file jci-133-172087-s218.pdf]

Supplemental data

Hemolysis Dictates Monocyte Differentiation via Two Distinct Pathways in Sickle Cell Disease Vaso-occlusion

Yunfeng Liu, Shan Su, Sarah Shayo, Weili Bao, Mouli Pal, Kai Dou, Patricia A. Shi, Banu Aygun, Sally Campbell-Lee, Cheryl A. Lobo, Avital Mendelson, Xiuli An, Deepa Manwani, Hui Zhong and Karina Yazdanbakhsh

Supplemental List:

Table S1

Figure S1

Figure S2

Figure S3

Figure S4

Table S1 Steady-state SCD patient information

Parameters	Patients with SCD
Number	n = 30
Age (years), median (min, max)	22 (14, 61)
Female	20 (67%)
Hemoglobin S (%), median (min, max)	72.4 (25.6, 91.1)
Post-splenectomy	2 (7%)
WBC ($x10^3/\mu l$), median (min, max)	9.55 (4.1, 17.3)
Neutrophil ($x10^3/\mu l$), median (min, max)	5.28 (2, 12.4)
Lymphocyte ($x10^3/\mu l$), median (min, max)	2.73 (1.32, 6.1)
Monocyte ($x10^3/\mu l$), median (min, max)	0.60 (0.17, 1.06)
Hemoglobin (g/dl), median (min, max)	9.05 (6.8, 13.3)
Reticulocyte ($x10^3/\mu l$), median (min, max)	293.8 (137.2, 675.2)
Platelet ($x10^3/\mu l$), median (min, max)	364 (181, 748)

Of the 30 patients: 13 (43%) were on a chronic transfusion protocol alone; 3 (10%) on a combination of hydroxyurea (HU) and chronic transfusion; 11 (37%) patients were on HU alone, 1 (3%) patient on Endari alone, and 2 (6.7%) patients on Voxelator alone; 7(23%) patients had a previous history of alloantibodies.

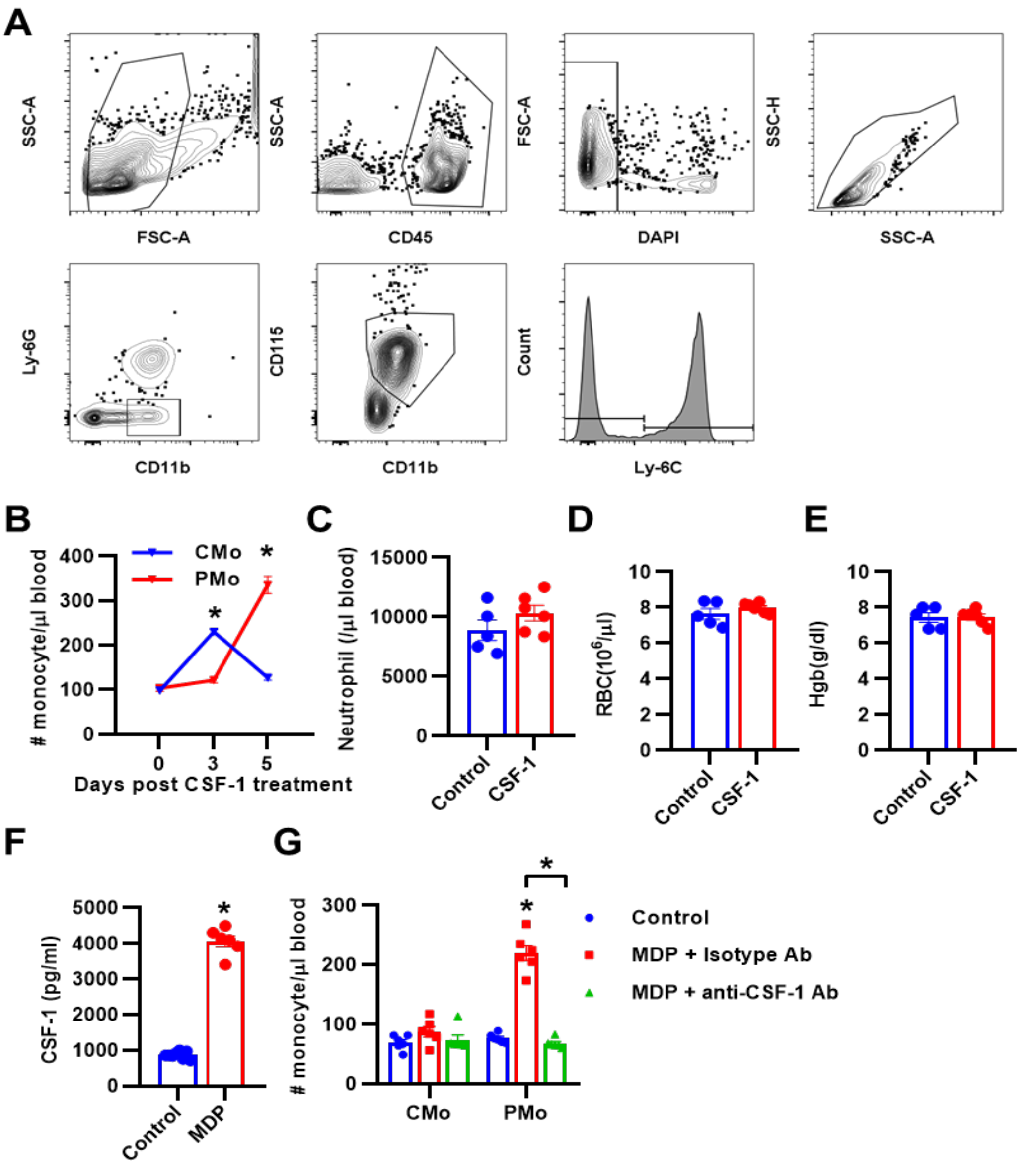

Figure S1 Monocyte subpopulations in mouse blood and response to CSF-1 and MDP treatments. (A) Gating strategy for mouse blood classical monocytes (CMo, CD11b+CD115+Ly-6C^{hi}) and patrolling monocytes (PMo, CD11b+CD115+Ly-6C$^{lo/-}$) in single live CD45+ leukocytes. (B) Absolute number of circulating Ly-6C^{hi} CMo and Ly-6C$^{lo/-}$ PMo in WT mice (n = 6) at day 3 and 5 post s.c. injection with CSF-1 (0.5 mg/kg body weight/day) or PBS control (200 µl/ 20 g body weight/day). (C) Blood neutrophil numbers, (D) Blood RBC numbers, and (E) blood hemoglobin (Hgb) levels in sickle mice at day 5 post s.c. injection with CSF-1 (0.5 mg/kg body weight/day) as in figure 1C (n = 5-6). (F) Plasma CSF-1 levels in WT mice at 20 hours after i.v. injection with MDP (1 mg/kg body weight) or PBS as control (n = 6-8). (G) Absolute number of circulating Ly-6C^{hi} CMo and Ly-6C$^{lo/-}$ PMo in WT mice at day 5 post treatment with MDP (1 mg/kg body weight/day) and blocking antibody against CSF-1 or isotype control (1 mg/kg body weight/two days) (n = 6). Symbols represent data from individual mice. Data are represented as mean ± SEM, and compared using a two-tailed Student's *t*-test in all the Figures except Figure B and G which were compared using two-way ANOVA with Bonferroni's multiple comparisons. * $p < 0.05$.

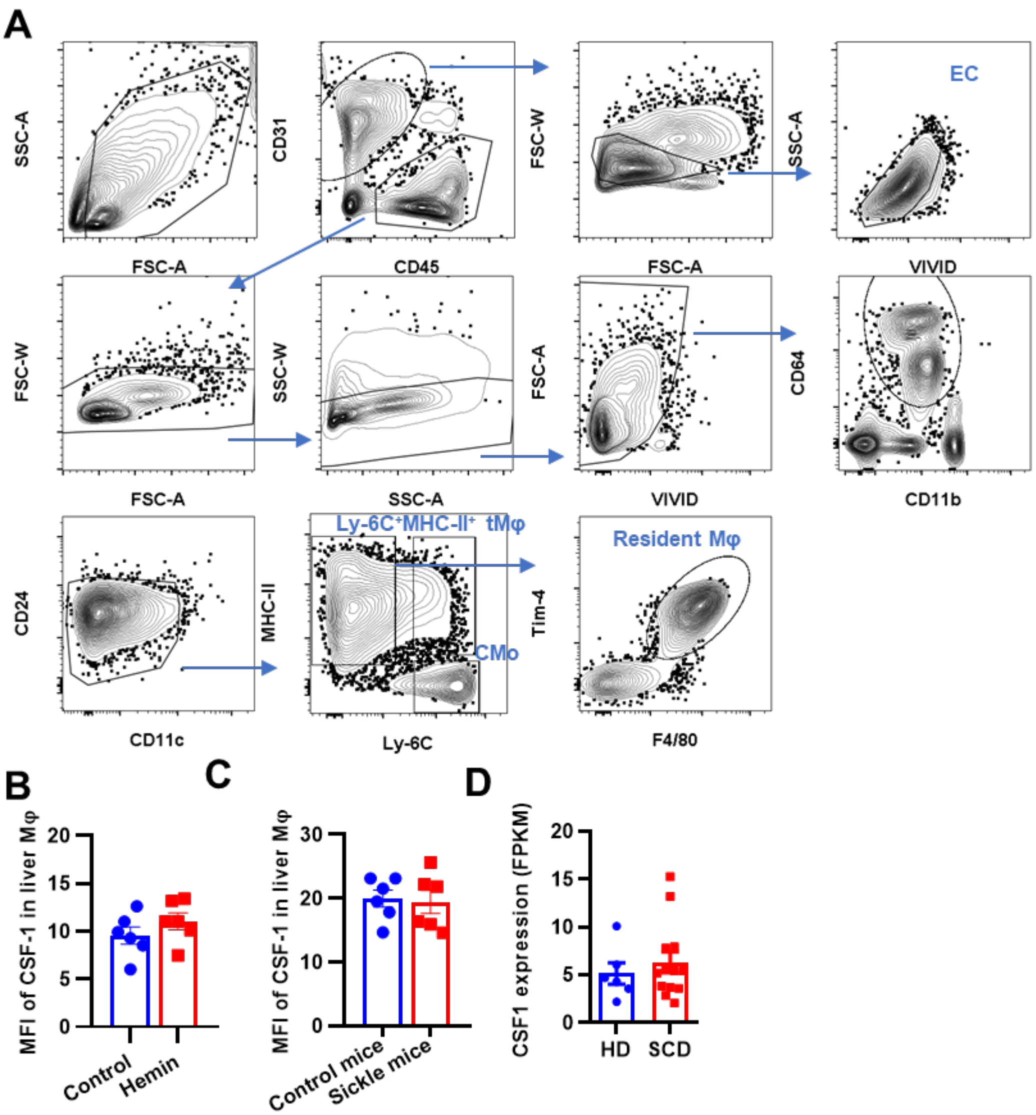

Figure S2 Mouse liver endothelial cell and leukocyte subpopulation gating strategy and CSF-1 expression. (A) Gating strategy for mouse liver endothelial cells (EC) as single ViViD$^-$CD45$^-$ CD31$^+$ cells, leukocytes as single ViViD$^-$CD45$^+$ cells, and leukocyte subsets including resident Mϕ as F4/80^{hi}CD11b^{lo}CD64$^+$Tim-4$^+$ CD11c$^{lo/-}$CD24$^{lo/-}$ leukocytes, CMo as Ly-6C^{hi}MHC-II$^-$ CD11b^{lo}CD64$^+$CD11c$^{lo/-}$CD24$^{lo/-}$ leukocytes, and Ly-6C$^+$MHC-II$^+$ transient macrophage (tMφ) as Ly-6C$^+$MHC-II$^+$CD11b^{lo}CD64$^+$CD11c$^{lo/-}$CD24$^{lo/-}$ leukocytes. (B) scatter plot with bar showing CSF-1 expression in liver resident Mϕ from WT mice at 20 hours post treatment with PBS as control (200 µl/ 20 g body weight) or hemin (35 µmol/kg body weight) (n = 6). (C) scatter plot with bar showing CSF-1 expression in liver resident Mϕ from sickle mice or control mice (n = 6). (D) CSF-1 expression in human CMo from SCD patients (n=13) and HD (n=6) (GSE149050, as in our published report[3]). Symbols represent data from individual mice. Data are represented as mean ± SEM, and compared using a two-tailed Student's *t*-test. * $p < 0.05$.

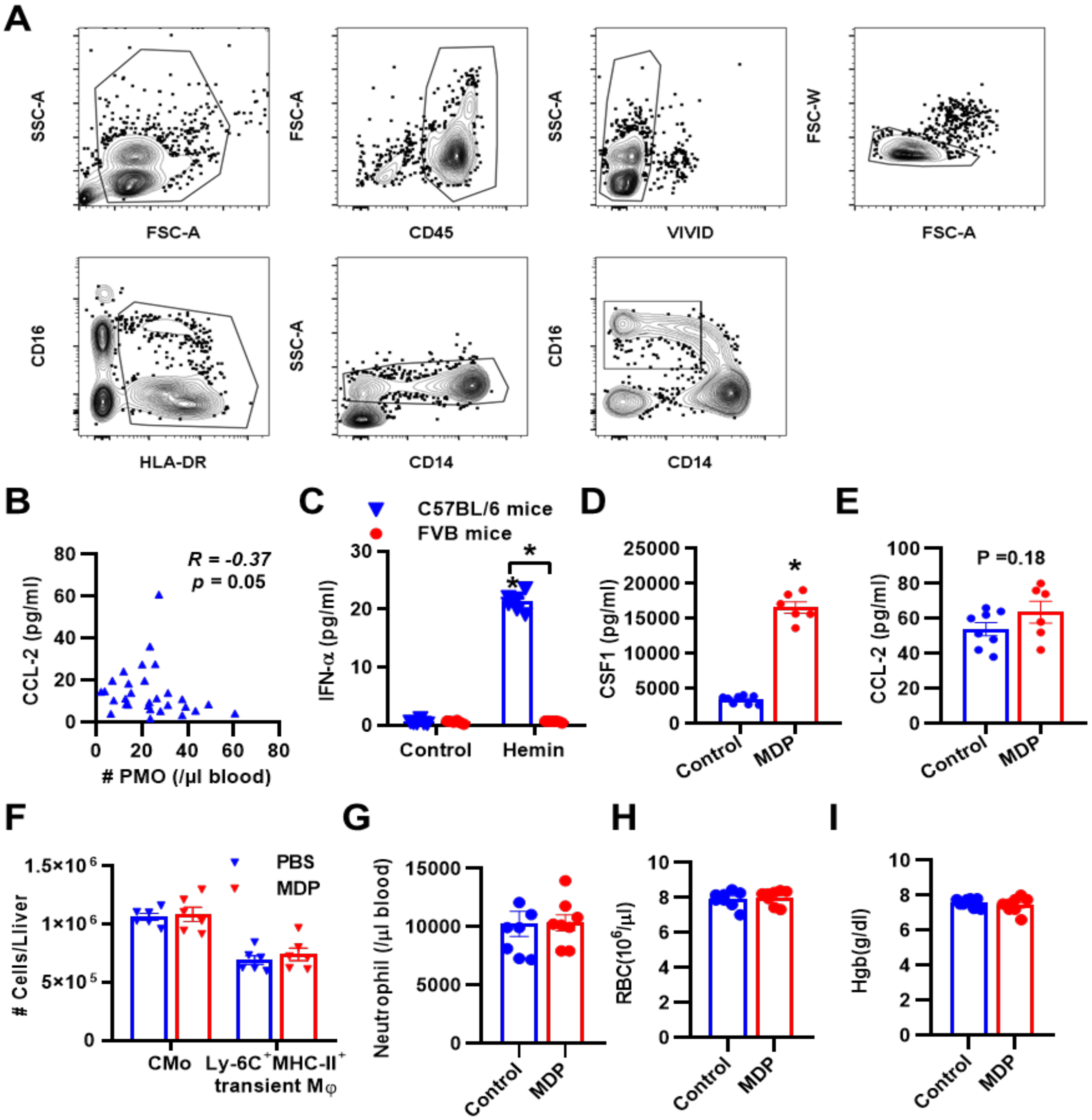

Figure S3 Correlation between plasma CCL-2 levels and circulating PMo numbers, and response to hemin and MDP treatments. (A) Gating strategy for human blood patrolling monocytes (PMo, HLA-DR$^+$SSC^{lo}CD14$^{lo/-}$CD16$^+$) in single live CD45+ PBMCs. (B) Scatter plot analysis showing correlation relationship between plasma CCL-2 levels and absolute numbers of circulating PMo in patients with SCD (n = 30 as in Fig. 1). (C) Plasma IFN-α levels in WT C57BL/6 mice and FVB mice 20 hours after i.v. injection with hemin (35 µmol/kg body weight), or PBS (200 µl/ 20 g body weight) as control (n = 6). Plasma CSF-1 levels (D) and CCL-2 levels (E) in sickle mice (n = 6-8) one day after treatment with MDP (1 mg/kg body weight) or PBS control. (F) Absolute number of liver Ly-6C^{hi}MHC-II$^-$ CMo and Ly-6C$^+$MHC-II$^+$ transient macrophage (Mφ) in sickle mice treated with MDP or PBS (n = 6) as shown in D. (G) Blood neutrophil numbers, (H) Blood RBC numbers, and (I) blood hemoglobin (Hgb) levels in sickle mice treated with MDP as shown in D (n = 8). The correlation analysis in (B) was determined by Spearman Rho. Symbols represent data from individual mice. Symbols represent data from individual mice. Data are represented as mean ± SEM, and compared using a two-tailed Student's *t*-test in all the Figures except Figure C and F which were compared using two-way ANOVA with Bonferroni's multiple comparisons.. * $p < 0.05$.

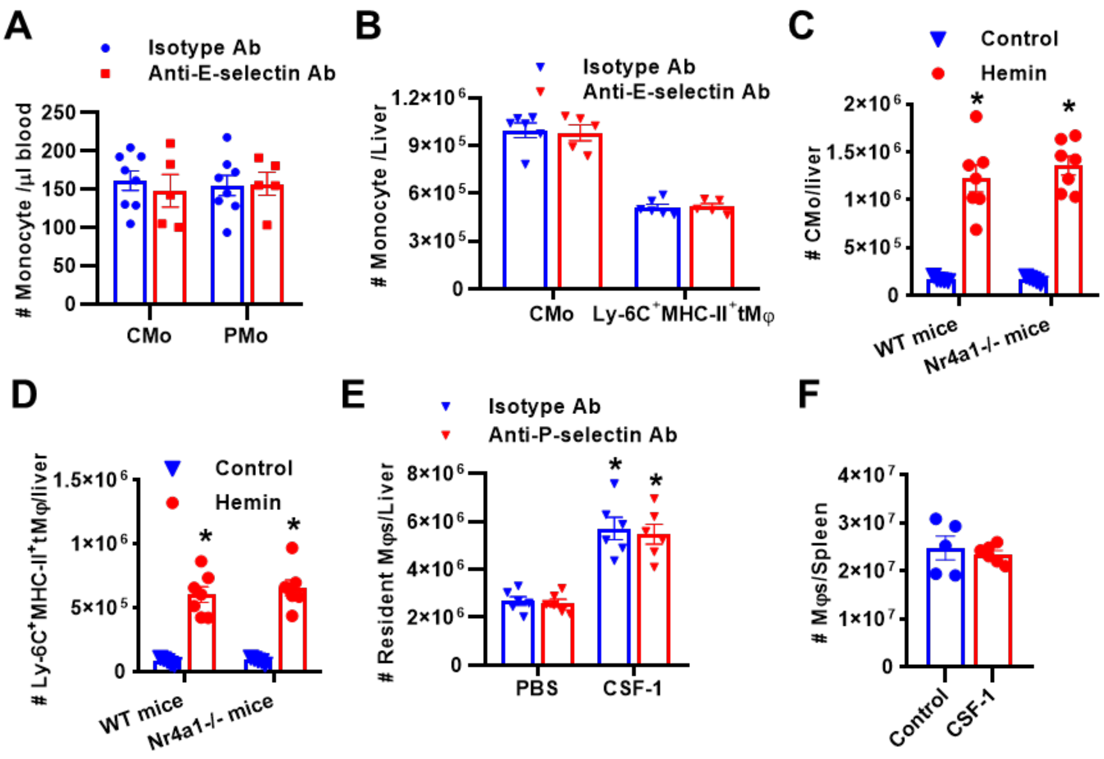

Figure S4 Response to hemin, CSF-1 and blocking antibodies treatments in mice. (A) Bar graph showing absolute number of circulating Ly-6C^{hi} CMo and Ly-6C$^{lo/-}$ PMo at 20 hours in hemin (35 µmol/kg body weight)-injected WT mice pretreated for 30 minutes with anti-E-selectin blocking antibody (5 mg/kg body weight, i.v.), or isotype control antibody (5 mg/kg body weight, i.v) (n = 5-8). (B) Bar graph showing absolute number of liver Ly-6C^{hi}MHC-II^{-} CMo and Ly-6C^{+}MHC-II^{+} transient macrophage (tMφ) in mice treatment as in A (n = 5-6). Absolute number of liver Ly-6C^{hi}MHC-II^{-} CMo (C) and Ly-6C^{+}MHC-II^{+} transient macrophage (tMφ) (D) in WT mice and Nr4a1$^{-/-}$ mice 20 hours after i.v. injection with hemin (35 µmol/kg body weight), or PBS (200 µl/ 20 g body weight) as control (n = 5-7). (E) Bar plot showing absolute number of liver resident macrophage (Mφ) in sickle mice at day 5 post anti-P-selectin blocking antibody or isotype control antibody (5 mg/kg body weight, i.p. every other day), with or without s.c. injection with CSF-1 (0.5 mg/kg body weight/day) as shown in Fig. 5 (n = 6). (F) Bar plot showing absolute number of spleen macrophage (Mφ) in sickle mice at day 5 post s.c. injection with CSF-1 (0.5 mg/kg body weight/day) or PBS as control (n = 5-6). Symbols represent data from individual mice. Data are represented as mean ± SEM, and compared using two-way ANOVA with Bonferroni's multiple comparisons in Figure A-E, and using a two-tailed Student's *t*-test in Figure F. * $p < 0.05$.
